# The Geographic Distribution, Venom Components, Pathology and Treatments of Stonefish (*Synanceia* spp.) Venom

**DOI:** 10.3390/md19060302

**Published:** 2021-05-24

**Authors:** Silvia L. Saggiomo, Cadhla Firth, David T. Wilson, Jamie Seymour, John J. Miles, Yide Wong

**Affiliations:** 1 Australian Institute of Tropical Health and Medicine (AITHM), James Cook University, Cairns 4878, Australia; cadhla.firth@jcu.edu.au (C.F.); david.wilson4@jcu.edu.au (D.T.W.); jamie.seymour@jcu.edu.au (J.S.); john.miles@jcu.edu.au (J.J.M.); yide.wong@jcu.edu.au (Y.W.); 2 Centre for Molecular Therapeutics, James Cook University, Cairns 4878, Australia; 3 Centre for Tropical Bioinformatics and Molecular Biology, James Cook University, Cairns 4878, Australia

**Keywords:** stonefish, *Synanceia*, venom, toxins, pharmacology, distribution

## Abstract

Stonefish are regarded as one of the most venomous fish in the world. Research on stonefish venom has chiefly focused on the in vitro and in vivo neurological, cardiovascular, cytotoxic and nociceptive effects of the venom. The last literature review on stonefish venom was published over a decade ago, and much has changed in the field since. In this review, we have generated a global map of the current distribution of all stonefish (*Synanceia*) species, presented a table of clinical case reports and provided up-to-date information about the development of polyspecific stonefish antivenom. We have also presented an overview of recent advancements in the biomolecular composition of stonefish venom, including the analysis of transcriptomic and proteomic data from *Synanceia horrida* venom gland. Moreover, this review highlights the need for further research on the composition and properties of stonefish venom, which may reveal novel molecules for drug discovery, development or other novel physiological uses.

## 1. Introduction

There are five species of stonefish within the genus *Synanceia*: *Synanceia horrida* (previously referred to as *S. trachynis*), *S. verrucosa*, *S. alula, S. nana* and *S. platyrhyncha*. *Synanceia* spp. can grow 35–50 cm in length and have evolved grey and mottled skin to camouflage themselves amongst encrusted rocks and coral for predation and defense [1]. *Synanceia* species have up to 15 dorsal fin spines that are erected when the fish is disturbed [2]. Stings from this medically important group of fish are known to cause painful and lethal human envenomations.

Although clinical reports of stings and injuries from *Synanceia* species have been reported since the end of the 19th century [3], studies into their venom biology, chemistry and pharmacology have been scarce, and the mechanisms of action of the venom remain mostly unknown. Fortunately, much has changed in this field in recent years due to technological advances, which have spurred new avenues of research. This review attempts to comprehensively summarize and update the current state of knowledge of *Synanceia* spp., with a focus on geographic distribution, human impact, disease burden, venom composition and the mechanisms of action of venom components.

## 2. Disease Burden of Stonefish Envenomation

Statistics from independent retrospective clinical studies of stonefish stings from hospitals in the Indo-Pacific region showed that most envenomations occurred in young adult males with occupational or recreational exposure to stonefish habitat [4,5,6,7]. Stonefish were the second most common fish involved in human envenomations in Hong Kong [5] and were responsible for one-third of all fish envenomations in Australia in 2008 [7]. A recent report by the Australian Institute of Health and Welfare stated that of all hospitalizations due to injury from contact with venomous marine plants and animals from 2017–2018, 81% were caused by stinging fish, where 30 out of 320 cases (9.3%) were due to stonefish envenomation [8]. Additionally, stonefish have been responsible for a high number of stings in Singapore [9,10], and there are concerns that the number of stings will grow in Japan due to warmer ocean temperatures and increasing tourism [6]. These studies also report that victims usually sought medical assistance within 2 h of being stung due to intense pain [4,6]. The majority of the cases were regarded as mild envenomation, with average hospital stays ranging from hours [4] to days [11]. In more severe cases, hospitalizations can last for over a week [6], and surgical interventions may be necessary in some instances [4,9,11].

While these reports highlight the burden of fish stings in the Indo-Pacific region, stonefish envenomation is grossly underreported in the literature [4]. This makes it difficult to precisely quantify the number of victims and the global impact of stonefish envenomation on an annual basis. Another confounder is that the diagnosis of a stonefish sting usually relies on the presence of puncture wounds and the clinical and systemic features that follow envenomations. Only rarely is the animal brought to the medical center, hindering the actual identification of the offending animal [11]. Stonefish envenomation case reports from literature to date are reviewed and summarized in Table A1. Caution in interpreting these numbers is further advised as generally, while case reports may state that the victim was stung by a *Synanceia* species, there is frequently no confirmation or validation of which marine creature caused the sting [4]. To date, there has been no research comparing or contrasting the effects of venom from different species of stonefish following envenomation. Thus, species differentiation by clinical presentation is currently not viable, and it is not known if venom effects differ across the genus.

## 3. Geographic Distribution of *Synanceia* spp.

**Figure 1:**
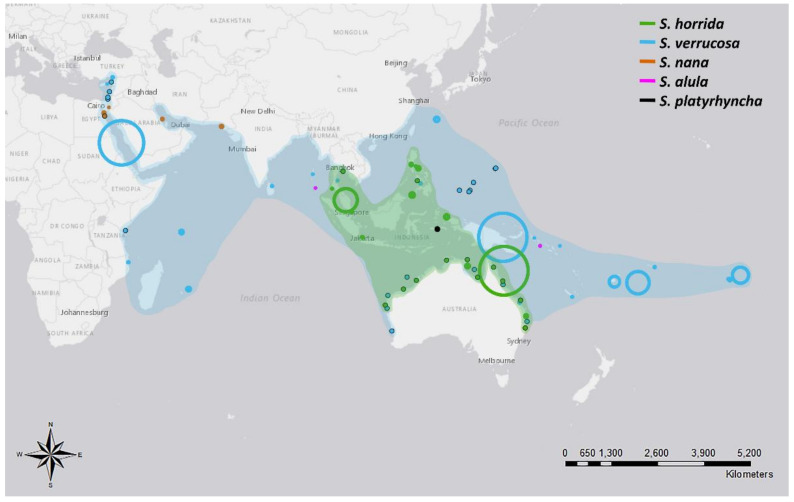
Distribution of five different species of stonefish of the genus *Synanceia*. Points with black outlines represent sightings where coordinates were provided. Circle size depicts the accuracy of the reported location; for example, large circles depict reports where only the country name was given, and small circles depict reports where the precise location was stated. Shades indicate the hypothesized distribution of both *S. horrida* and *S. verrucosa* based on collated sighting data. Animals would be found in the shallow regions within the shaded areas. References listed in Table A2 (Figure generated with ArcMap 10.7 and Photoshop 2021).

Species of the *Synanceia* genus have a broad overlapping distribution throughout the shallow waters of tropical and temperate areas of the Indo-Pacific (Figure 1 and Table A2). *S. verrucosa* (reef stonefish) has the broadest distribution, with confirmed sightings off the coasts of Turkey, Mozambique, Sri Lanka, Singapore, Japan and Australia and throughout the islands of the Pacific Ocean to French Polynesia. *S. horrida* (estuarine stonefish) has the second largest distribution, with confirmed sightings from mid to northern Australia and throughout Malaysia, Indonesia, Singapore, Thailand and the Philippines. *S. nana* appears to be confined to the waters of the Persian Gulf and the Red and Arabian Seas, whereas *S. alula* and *S. platyrhyncha* have been observed only occasionally (Figure 1 and Table A2). Interestingly, only one holotype of *S. platyrhyncha* was located, and upon further examination, Eschmeyer and Rao (1973) stated that *S. platyrhyncha* may be *S. horrida*, but more specimens were required for validation. Although there have been some suggestions of *Synanceia* species present in the Florida Keys and the Caribbean Sea [12], no further evidence was found in the literature to support this.

**Figure 2:**
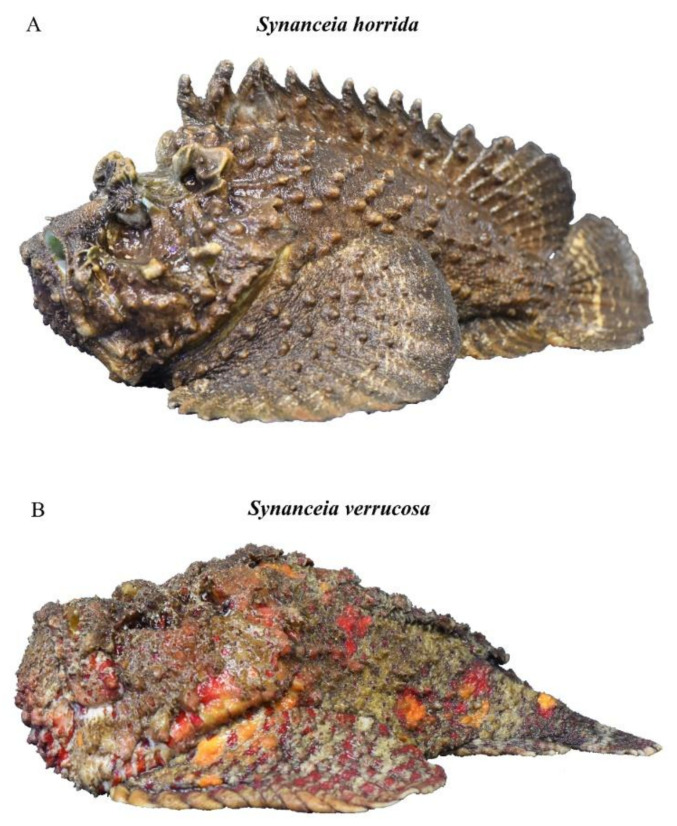
Side profiles of (**A**) *Synanceia horrida* and (**B**) *Synanceia verrucosa* (Photo: Jamie Seymour).

Although there are five species of *Synanceia*, only two have been extensively studied: *S. horrida* and *S. verrucosa*. These species are considered the world’s most venomous fish species [3]. *S. horrida* has 13 to 14 dorsal venom spines and is commonly found in estuaries, sheltered bays, shoal reef areas and tide pools [2,13] (Figure 2A). The head is depressed and large, and the most distinguishable features are the deep pits below the eyes and the eyes in elevated stalks with high crests joining them [2]. *S. verrucosa* can have 12 to 14 dorsal venom spines and is commonly found in coral reef areas [3,13] (Figure 2B). The head is broad, large and depressed, and the eyes are marginally elevated [2]. Both species are sluggish, ambush predators that often sit motionless and partially buried in the substrate [13,14].

## 4. Envenomation

### 4.1. Venom Apparatus

**Figure 3:**
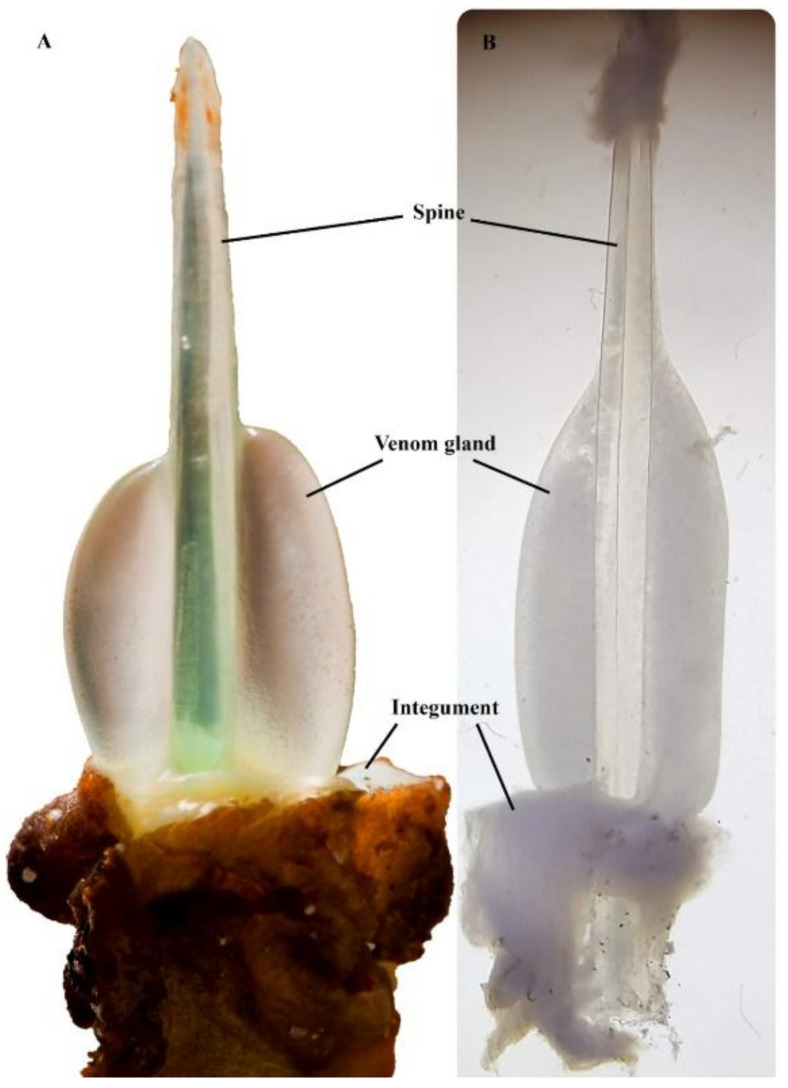
*Synanceia* venom glands: (**A**) the thick integument sheath was removed from the second spine (longest) of *S. horrida*, revealing the venom gland in situ (Photo: Jamie Seymour); (**B**) venom gland dissected out from the last spine (shortest) of *S. verrucosa*, under the microscope (Photo: Silvia Luiza Saggiomo). Pictures are not to scale.

One of the remarkable physical attributes of stonefish is their grooved hypodermic-like dorsal spines (Figure 3). These spines are used purely as a defensive mechanism, as they are erected by the animal when threatened [15]. Each dorsal spine is associated with a pair of venom glands, which are covered by a loose and thick integumentary sheath [16]. When force is applied vertically on the dorsal spines, the integument is ruptured, and the surrounding tissue of the predator or the victim compresses the dual venom glands, involuntarily releasing venom through the spinal venom duct [16]. Each pair of dorsal venom sacs contains approximately 5–10 mg of dried venom [17]; thus, the severity of symptoms is typically associated with the number of spines involved in envenomation and the depth of spine penetration [10].

### 4.2. First Aid, Clinical and Systemic Features of Stonefish Stings

The pain associated with stonefish stings is intense, excruciating, disproportionate to the size of the injury and may spread to include the whole limb and associated lymph glands [3]. The envenomed limb displays puncture wounds with a blue coloration, gross edema and local morbidity [11]. Successful first aid for fish stings usually focuses on pain alleviation, treatment of the injury site and the effects of envenomation [18]. Although there seems to be no consensus on venomous fish sting first aid, hot water therapy is recommended as an effective pain management tool [19,20]. For stonefish envenomation in particular, it has been suggested to keep the affected limb immersed in water at 42 °C for at least 20 min to inactivate the venom [21]. Victims should also seek medical aid, where treatments might include radiography, ultrasound, debridement of the wound, local anesthetics, tetanus prophylaxis and administration of stonefish antivenom [11,16,22]. Systemic symptoms include fever, delirium, muscle weakness and paralysis, pulmonary edema, respiratory difficulties, hypotension, bradycardia, arrhythmia, convulsions, heart failure and death [16,22,23,24,25].

In recent years, several clinical reports of stonefish envenomation indicate that victims may have lingering complications after being stung (Table A2). Some symptoms, such as asthenia and trophic disorders, may persist for months in the affected limb, and authors recommended ongoing care of the puncture site and broad-spectrum antibiotics for a few days after envenomation [11,26,27]. Additionally, acute compartment syndrome due to edema has been reported in stonefish sting victims; thus, surgical procedures may be required to avoid permanent neurological damage [28,29,30], and amputations due to necrosis have been necessary in some cases [31,32,33]. Deaths caused by stonefish envenomation have been the subject of great debate, as specific details of the recorded deaths have been poor or missing altogether [16,34]. Unfortunately, a recent case report discussing three cases of stonefish envenomation described the death of an 11-year-old boy after being stung in the foot in 2018 [27]. It appears, therefore, that stonefish envenomation causes serious symptoms and pathologies, where long-lasting effects may affect victims worldwide, and lethal envenomations may in fact occur.

### 4.3. Antivenom

*Synanceia* antivenom works by neutralizing the hemolytic, lethal and vascular permeability-increasing properties of the venom [15,35]. The efficacy of stonefish antivenom has been demonstrated both in vitro and in vivo [15,35], and its clinical efficacy at combating venom-induced tissue damage and providing analgesia is well established [26,35,36,37]. Antivenom can be injected intramuscularly or administered through intravenous infusion in severe cases [22]. An ampoule containing 2000 units of antivenom neutralizes approximately 20 mg of venom, and dosages are managed according to the number of puncture wounds present in the affected limb [38].

Interestingly, *Synanceia* antivenom has shown cross-reactivity with different species of venomous fish in both in vitro and in vivo models (Table 1). In particular, this antivenom appears to exhibit cross-reactivity with venoms from the South Australian cobbler (*Gymnapistes marmoratus*) and the red lionfish (*Piterois volitans*) in immunoblotting analysis [37]. This cross-reactivity may be explained by the close phylogenetic relationship between these species and *Synanceia* species [39], or perhaps these venoms and their constituents possess similar structures and/or modes of action [40]. Interestingly, the antivenom used for the aforementioned studies was raised against venom from *S. horrida* only (i.e., monospecific). Currently, however, stonefish antivenom is produced using both *S. horrida* and *S. verrucosa* venoms at Seqirus Pty. Ltd., Melbourne, Australia (Commonwealth Serum Laboratories) [16]. Therefore, it may be possible to create a polyvalent (i.e., polyspecific) antivenom that would be effective in treating stings from the most medically important species of venomous fish, increasing the antivenom efficacy, as seen with snake antivenoms [41].

## 5. Composition of *Synanceia* spp. Venoms

Lability is a key characteristic of stonefish venom. Stonefish venom has shown susceptibility to changes in pH, temperature, storage conditions, lyophilization and repeated freezing and thawing [45,46,47,48]. Additionally, there are reports of fish-to-fish variation in toxicity [49], and a recent study into *S. horrida* venom profile associated with different feeding regimes found that venom production is also affected by starvation [50]. Individual variation in venom composition has been shown in several different species, including snakes [51], jellyfish [52], spiders [53] and scorpions [54], which can affect the efficacy of the venom. Therefore, it is possible that other potential variables may influence venom composition. Factors such as geographical location, season, gender and age of fish may play a significant role; however, none of these have been investigated or reported for *Synanceia* venom yet. Not surprisingly, therefore, early attempts at purifying stonefish venom for experimental purposes were largely unsuccessful.

Progress in fish venom research has rapidly advanced with improvements and advances in biochemical separation processes and molecular analyses [17,55]. In particular, advances in fractionation, purification and isolation of the toxic elements in *Synanceia* venoms have revealed that all of the lethal activity appears to be caused by only a few toxins or proteins [49,56]. Stonustoxin (SNTX), isolated from *S. horrida* crude venom, was shown to be a dimeric protein consisting of an α- and β-subunit, each with a calculated molecular weight (MW) of 79 kDa (Table 2) [57]. SNTX has a p*I* of 6.9, can comprise as much as 9% of the total protein content of crude venom and has been shown to be 22-fold more toxic than crude venom [56] (Table 3). In addition, a cytolysin with a p*I* of 5.7 and a MW of 158 kDa has also been purified from *S. horrida* venom and was found to possess lethal activity and cell membrane-damaging properties in mice (Table 2) [58].

Verrucotoxin (VTX), isolated from *S. verrucosa*, is a tetrameric toxin (two α- and two ß-subunits) with a MW of 322 kDa (Table 2) [49]. Each α-subunit has a MW of 83 kDa, and each ß-subunit has a MW of 78 kDa. Less than 135 ng/g body weight of VTX is capable of causing immediate death in mice [49]. A proteic complex has also been isolated from VTX, termed p-VTX, which is more stable than VTX but exhibits no lethal or hemolytic activities [65]. Furthermore, a glycoprotein (neoVTX) that has both hemolytic and lethal properties has also been isolated from *S. verrucosa* venom [61]. Like SNTX, neoVTX is a dimeric protein with a MW of 166 kDa and is composed of two subunits, α- and ß-, each with a MW of 79 kDa by deduced amino acid sequence [61] (Table 2). Table 3 lists the different LD_50_ values for these toxic components or venom preparations in relation to the stonefish species of origin and route of delivery in anesthetized mice. The isolated toxins appear to be more potent than crude or reconstituted venoms and are believed to be responsible for much of the venom’s lethal and hemolytic activities.

Remarkably, no significant amino acid sequence similarity has been identified between *Synanceia* toxins and other animal toxin proteins, suggesting that *Synanceia* toxins are novel molecules [66]. Not surprisingly, however, significant sequence similarity has been found within the active proteins that comprise different stonefish venoms. For example, the VTX ß-subunit shares 96% amino acid sequence similarity with the SNTX ß-subunit across the first 72% of the protein sequence [66], and neoVTX α- and ß-subunits share 87% and 95% amino acid similarity with the SNTX α- and ß-subunits, respectively [61]. Interestingly, neoVTX appears to differ from SNTX only in the number of cysteine residues and free thiol groups, whereas it appears to differ considerably from the composition of VTX [61].

A comprehensive characterization of the venom gland transcriptome and the proteome of crude venom from *S. horrida* has recently been published [60]. This analysis revealed that the venom proteome is primarily composed of proteins identified as C-type lectins and SNTX, with additional putative hyaluronidase and peroxiredoxin present [60] (Table 2). C-type lectins appear to be the most abundant component in the proteome and are likely responsible for the hemagglutinating activity of the venom that contributes to the inflammation observed in envenomations [60]. Peroxiredoxins are antioxidant proteins believed to be involved with the functional and structural diversification of toxins through disulfide bond formation [73]. Hyaluronidases are found in the extracellular matrix of many different organisms, binding water molecules, metal ions and salts and functioning as an intercellular cement [74]. Hyaluronidase is found in human body fluids and organs, as well as in the venoms of scorpions, lizards, spiders and snakes, amongst other organisms [74]. Although hyaluronidases are not toxins *per se*, they are believed to act as spreading factors that mediate the diffusion of toxins throughout the body [75]. Purified hyaluronidase (SFHYA1) from *S. horrida* venom is a heat-labile glycoprotein with a p*I* of 9.2 and a MW of 62 kDa (Table 2), and it has not been associated with the lethal or hemorrhagic activities of venom [59]. SFHYA1 appears to be specific to hyaluronic acid (HA) [76] and resembles the PH-20 hyaluronidase family, which is a group of multifunctional proteins with unique enzymatic properties and expression patterns in different tissues [77]. Interestingly, the purified hyaluronidase showed a 261-fold increase in activity compared to the crude venom when measured by turbidimetric assays [59]. When a comparison was made between snake venom hyaluronidase and SFHYA1, SFHYA1 showed activity that was many-fold higher when hydrolyzing 50% of the HA. Further measurements showed that SFHYA1 was present at concentrations approximately 10^5^-fold higher in the estuarine stonefish venom than the hyaluronidases in snake venoms [59].

*S. horrida* venom also contains other enzymatic proteins, including acetylcholinesterase, alkaline phosphomonoesterase, phosphodiesterase, arginine amidase, 5′ nucleotidase, arginine ester hydrolase and proteases [60,69]. Analysis of the venom gland transcriptome found that the majority of the assembled contigs (59.3%) showed no homology to any existing protein in the Swiss-Prot database, while 40.3% were similar to nontoxic housekeeping genes responsible for cellular maintenance and function, and 0.4% were homologous to known venom components [60]. The percentage of contigs from the complete venom gland transcriptome of *S. horrida* found to be homologous to putative venom components was much lower compared to those found in the venom gland transcriptomes of other venomous species such as snakes (24–27%) and scorpions (53%) [60]. Additionally, a relatively low proportion of putative animal toxin families was identified in the venom, which might be explained by the defensive role the venom plays in the ecology of the animal, where a low diversity of distinct toxins would be sufficient to deter predators [60]. The low number of distinct toxins found in the venom could also be explained by mechanical damage to the venom gland during milking in this particular study, causing an upregulation of the proteins needed to rebuild and repair the gland itself relative to proteins responsible for the toxic activity of the venom [60]. Overall, the venom composition of *S. horrida* appears to be unique when compared to other venomous species, containing several proteins that have not been previously recognized in proteomic studies of other venoms [60].

Crude *S. horrida* venom also contains small molecules such as norepinephrine, dopamine and tryptophan, which are known regulators of cardiac physiology [49] and may play a role in the symptoms and pathologies experienced by sting victims [78]. In addition, negligible amounts of histamine, a potent inflammatory mediator associated with pain and edema, are present [79]. Investigation into whether or not stonefish venom elicits the release of endogenous stores of histamine found that this is unlikely, as it appears that the venom does not act on histamine receptors in guinea pig smooth muscles [79]. Interestingly, serotonin, which was previously thought to be linked to the pain caused by stonefish envenomation and contribute to bronchoconstriction and vasodilation, has not been detected in any *Synanceia* venom thus far, even though these symptoms are consistent with stonefish envenomation [78,79].

Fewer studies have been performed on the venom of *S. verrucosa*; however, a 59 kDa hyaluronidase (Table 2) has been partially purified and, like SFHYA1, shows activity only against HA. It was also the first fish hyaluronidase reported to act as a spreading factor [64]. Notably, although *S. verrucosa* hyaluronidase appears to be structurally and enzymatically similar to SFHYA1 with 92% amino acid sequence identity, it exhibits less than 50% identity with hyaluronidases from the honey bee or snake hyaluronidases, for example [64]. Other enzymes, including lipases and aminopeptidases, have also been identified in this venom [49]. Similar to *S. horrida*, *S. verrucosa* crude venom also contains norepinephrine, dopamine and tryptophan [78]. Apart from those, cardioleputin, a cardiotoxic 46 kDa protein (Table 2), has been isolated from *S. verrucosa* venom. It is primarily composed of glycine, serine and glucosamine, with few basic amino acids and no cystine [62]. Similar to the crude venom, cardioleputin toxicity can be lost at high temperatures, as well as during dilution and freeze-thawing [62]. Its cardiotoxic activity is described in the next section. *S. verrucosa* venom has also been shown to contain novel lectins, which are proteins that bind specific carbohydrates to mediate several different biological processes [80]. Experimental fractions from crude *S. verrucosa* venom named Con A-I and Con A-II were shown to have hemagglutinating and mitogenic activities, where Con A-I showed stronger activity compared to Con A-II and the unfractionated crude venom [63]. Further analysis of Con A-I revealed the presence of two subfractions, Con A-I-PS-I and Con A-I-PS-II [63]. The first subfraction contained three proteins (42.1, 100 and 110 kDa in MW), while the second showed only one protein of 45 kDa, named 45 kDa lectin (Table 2). Both Con A-I-PS-I and the 45 kDa lectin did not show toxicity to human leukemia cells (K562) [63].

## 6. Cardiovascular and Respiratory Effects of *Synanceia* Envenomation

While crude *S. verrucosa* venom has been shown to produce positive inotropic and chronotropic responses in isolated frog atrial fibers [81], the isolated compounds from *S. verrucosa* venom produce a range of reactions in vivo and in vitro. For example, purified VTX causes a marked dose-dependent hypotensive effect in anesthetized rats and cardiac and respiratory failure in mice in vivo [49]. In contrast, in vitro investigations have revealed that both VTX and p-VTX produce negative chronotropic and inotropic effects in frog atria, where p-VTX is postulated to open K^+^ channels during the cardiac cycle and reduce the amount of Ca^2+^ that enters the cells through competitive inhibition of Ca^2+^ binding sites [65]. VTX seemingly inhibits K_ATP_ currents in a voltage-independent and dose-dependent manner in guinea pig ventricular myocytes [82]. The reason for this discrepancy between the effects of VTX on frog and guinea pig hearts is unclear, but it is possible that these two species have different regulatory K_ATP_ pathways [82], as there seems to be a difference between mammalian ß_1_- or ß_2_-adrenoceptors and atrial amphibian ß-adrenoceptors [83]. Furthermore, when the effects of cardioleputin on guinea pig atria were investigated, irreversible positive inotropic and chronotropic effects were identified, which suggests lack of phospholipase A_2_ activity, as well as a possible action on atrial membrane Ca^2+^ channels [62], supporting the results of Sauviat et al. (1995).

Similarly, reconstituted *S. horrida* venom also appears to cause a range of effects depending on the envenomation model and the methods used. This venom seems to have potent myotoxic effects on cardiac, skeletal and involuntary muscles [84]. These effects can be observed following intravenous injection of reconstituted venom in anesthetized rabbits, where muscular paralysis, respiratory distress and hypotension are observed [84]. In contrast, reconstituted venom appears to cause a biphasic response in anesthetized rats, where the initial pressor response is partly mediated by α_1_-adrenoceptors and leukotriene receptors, and the depressor response is mediated by β_2_-adrenoceptors, creating an overall hypertensive effect [85]. The reasons for these discrepancies are not yet understood.

In vitro studies using purified SNTX on rat aortic rings have demonstrated potent vasorelaxant activity [86]. This vasorelaxation effect appears to be mediated by an endothelium-dependent mechanism, most likely facilitated by the L-arginine–nitric oxide synthase pathway [86]. Endogenous hydrogen sulfide (H_2_S) acts in synergy with nitric oxide (NO) [87] to bind to endothelial substance P neuropeptide receptors, producing NO and activating K^+^ channels, ultimately leading to the SNTX-induced muscle relaxation observed [88]. Furthermore, SNTX was found to contain a B30.2 domain, which is present in multiple intracellular, transmembrane and secreted proteins [89,90]. This domain was shown to indirectly increase NO production through competitive binding of the NO synthase inhibitor, xanthine oxidase, in bovine cerebellum, which might explain the role of NO and the SNTX-induced muscle relaxation [90].

The respiratory failure observed in experimental *Synanceia* envenomation may also contribute to the lethal activity of stonefish venom [86]. Respiratory failure caused by *S. horrida* envenomation could ensue from direct skeletal muscle paralysis, as neuromuscular conduction through the phrenic nerve–diaphragm junction is not inhibited even when respiration has stopped in anesthetized rabbits [84]. In the case of *S. verrucosa* venom, irregular or weakened respiration is observed, often followed by cessation of respiration when lethal doses of crude venom or VTX are administered in anesthetized rats [49]. Furthermore, pathological activity can be observed in mouse lung after intramuscular injections of *S. verrucosa* crude venom [71]. Apart from these experimental envenomation studies, a case report described three instances where patients were admitted to hospital after being envenomed by *S. verrucosa*. One of the victims required CPR and continued to show dyspnea and bilateral rattles, where acute pulmonary edema was hypothesized, although not confirmed [27]. In two other cases, pulmonary hypertension and acute pulmonary edema were diagnosed, evidenced by interstitial edema and bilateral pleural effusions. In one of those instances, the pathologies were severe, where the patient also suffered from cardiorespiratory arrest, left ventricle dysfunction and pericardial and pleural effusions and did not survive [27].

### G-Protein-Coupled Receptors

G-protein-coupled receptors (GPCRs) are one of the largest families of membrane proteins [91]. This family mediates the majority of physiological responses, including neurotransmission and hormonal responses, amongst other functions [91]. *S. verrucosa* venom appears to act on several GPCRs. For example, when examining venom effects in guinea pig ventricular myocytes, VTX appeared to modulate ion channels by stimulating the ß-adrenoceptor–cyclic adenosine monophosphate–protein kinase A (cAMP-PKA) pathway [92]. When stimulated by agonist binding, this ß-adrenoceptor leads to increased cAMP production via G-protein dissociation and adenylyl cyclase activation. The cAMP signaling then activates PKA, causing phosphorylation of several proteins downstream of this cascade, including L-type Ca^2+^ channels, which promotes an inotropic effect in cardiomyocytes [93].

Reconstituted crude *S. horrida* venom also appears to act on bradykinin receptors. A ß_2_-receptor antagonist, acting as a noncompetitive inhibitor of bradykinin, was shown to inhibit the relaxation response induced in pig coronary arteries by reconstituted crude *S. horrida* venom or bradykinin [48,94]. Bradykinins are peptides involved in several pathophysiological processes [95], such as inflammation, pain, increase in capillary permeability and vasodilation and decrease in vascular resistance [96]. Therefore, some cardiovascular symptoms might be explained by the venom-induced effects on bradykinin receptors and may also contribute to the excruciating pain experienced by victims [48]. In addition to bradykinin stimulation, *S. horrida* venom also seems to act on muscarinic receptors by stimulating endogenous acetylcholine production or mimicking its effect on the cardiac membrane [48,69,97]. When porcine coronary arteries were exposed to venom, a cholinergic-like endothelium-independent contraction was observed. Atropine, an antimuscarinic drug, inhibited this effect, which further supports the suggestion that *Synanceia* venom has some action at muscarinic receptors [48]. Research supporting this finding showed that purified SNTX induces the release of acetylcholine from atrial nerve terminals in frog atrial fibers, indirectly activating muscarinic receptors [97]. Given what is known about muscarinic receptors and the physiological effects of their activation, the responses observed in both experimental models and clinical reports suggest that muscarinic activity is a significant contributor to the cardiovascular pathophysiology of *Synanceia* envenomation. VTX can also stimulate muscarinic receptors and activate Ca^2+^ currents, leading to arrhythmia and hypoxia, preventing the activation of cardioprotective K_ATP_ currents and promoting cardiovascular collapse [82,92]. VTX does not seem to inhibit K_ATP_ current through activation of adenosine receptors or α_1_-adrenoceptors. Instead, it was strongly suggested that VTX activates the M_3_ receptor–protein kinase C (PKC) pathway [82].

There is evidence that venom from *S. horrida* stimulates the release of endogenous tachykinins. *S. horrida* venom-induced bronchoconstriction responses were reduced by an NK_1_ receptor antagonist in anesthetized guinea pigs in vitro [85], indicating that the venom acts on NK_1_ receptors possibly through the release of substance P (SP) [79]. SP is an important neurotransmitter released from nerve endings and transmits nociceptive signals by immune cells and various non-neuronal cells [98]. The venom may also further stimulate the endogenous release of cyclooxygenase products, which play an important role in increasing the activities of pain-producing inflammatory mediators and acetylcholine [79].

## 7. Vascular Permeability and Cytolytic Effects of *Synanceia* Envenomation

Crude *S. horrida* and *S. verrucosa* venoms produce potent inflammatory responses in in vivo studies and in patients, where stonefish victims show signs of tendon inflammation, pulmonary edema and other cardiac complications [26,27,99]. Research shows that *S. horrida* venom causes edema when administered intradermally into rat hind paws and mouse footpads [58,69,100]. The increase in vascular permeability associated with *Synanceia* envenomation does not appear to be triggered by histamine release because diphenhydramine, an antihistamine, does not seem to have an effect on the edema-inducing properties of SNTX [56]. Instead, it has been suggested that stonefish hyaluronidase might be responsible, at least in part, for this activity as it enhances the capillary-increasing activity of neoVTX when co-injected intradermally in mice [64].

Crude venoms from *S. verrucosa* and *S. horrida*, as well as VTX and SNTX, exhibit strong lytic actions against diluted blood and washed erythrocytes of a variety of mammal species [49,56,58,69]. Crude venom from *S. horrida* does not cause hemorrhage or have any measurable dermonecrotic effects in mice but seems to possess some anticoagulant activity in rabbit blood [69]. Additionally, reconstituted crude *S. horrida* venom appears to prevent clotting of human fibrinogen when Ca^2+^ is present, and this anticoagulant activity seems to be concentration-dependent [69]. When examining the effects of SNTX on whole blood, some hemolysis and platelet aggregation were observed in vitro in blood collected from rats and rabbits [101]. Notably, no hemolytic or coagulation modulating properties were observed in mouse or human studies, although *S. horrida* venom appears to cause cell lysis in cultured murine cortical neurons [42]. This lack of lytic activity in mouse erythrocytes may indicate that the direct cause of death in experimental animals may not be due to hemolysis [56].

Apart from these, thromboelastography assays were performed using fresh and lyophilized *S. verrucosa* venom on recalcified human plasma [47]. Although fresh venom did not have an effect on clot strength, it exhibited anticoagulant properties, demonstrated by a delay in time until clot formation. Lyophilized venom, in turn, did not show significant anticoagulant activity [47]. Fibrinogen levels can be assessed by clot strength and, since clot strength was not affected, the anticoagulation activity observed likely occurred upstream in the clotting cascade instead of being directly related to fibrinogen cleavage [47]. Further testing could not unravel the mechanism of action, but Harris et al. [47] suggested the venom possibly degrades phospholipids, which would ultimately lead to the anticoagulation seen [47].

The observed hemolytic activity produced by *Synanceia* venom in some animals has been linked to the formation of pores in cell membranes [102,103]. Experiments on the function and structure of mice kidneys using intramuscular injections of crude *S. verrucosa* venom resulted in elevated levels of the oxidative stress marker malonaldehyde caused by lipid peroxidation, which indicates that venom-induced damage to the cell membrane may be caused by the generation and action of free radicals [104]. Moreover, the SNTX complex of *S. horrida* venom, in particular, is one of the largest naturally occurring toxins isolated to date that possess pore-forming activity [102]. SNTX subunits belong to the perforin superfamily of pore-forming immune effectors [105]. Each SNTX protein has four domains, where the *N*-terminal domain is homologous to the membrane attack complex–perforin/cholesterol-dependent cytolysin (MACPF/CDC) pore-forming domains [105]. Proteins from the MACPF/CDC superfamily are typically promiscuous, suggesting that SNTX may form pores in a variety of tissues, likely having a central role in SNTX-induced pathologies and physiological effects [105]. Interestingly, it was suggested that SNTX binds to the membrane irreversibly, where a certain amount of toxin is presumably required to change membrane permeability [106]. Additionally, a smaller protein has been isolated from the crude venom of *S. horrida* that is homologous to perforin-1-like proteins found in several teleost fish species [60]. This protein has a C2 domain that targets cell membranes and binds to phospholipids [60], which constitutes the majority of the lipid components in mammalian membranes [107], and may further enhance the venom’s pore-forming cytolytic activities [60].

To understand the mechanism behind the toxicity of *Synanceia* venoms and the functional structure of their isolated fractions, inactivation studies have been performed with SNTX and neoVTX. The hemolytic activity from both neoVTX and SNTX is inhibited by modified anionic lipids [61,102]. Furthermore, SNTX’s hemolytic activity is also inhibited by the presence of some anionic lipids and chemical modification of tryptophan, thiol groups and lysine and arginine residues within the protein [102,108,109,110]. Additionally, SNTX’s lethal properties are inhibited by chemical modification of lysine residues, thiol groups and some cationic amino acids in the protein [102,108,109]. Interestingly, the hemolytic and lethal activities of SNTX might not originate from the same domain or region of the protein [109], as the lethal activity is lost after lyophilization or temperature changes, but the hemolytic activity is retained [109,111].

## 8. Neuromuscular Effects of *Synanceia* Envenomation

Experiments to determine the neuromuscular effects of crude *S. horrida* venom have demonstrated that the venom may also block neurotransmitter synthesis, ultimately leading to a depletion in neurotransmitter stores [69]. When applied to extracted mouse hemidiaphragms, *S. horrida* venom can cause irreversible neuromuscular blockages, which may lead to fatal respiratory paralysis [112]. Interestingly, SNTX irreversibly blocks both nerve- and muscle-evoked twitches of mouse isolated nerve–hemidiaphragm and chick biventer cervicis muscle preparations [113]. This inhibitory action of SNTX on neuromuscular function was likely the result of direct myotoxic effects of SNTX in the muscle as the contractile responses to acetylcholine, carbachol and potassium chloride were completely blocked [113]. SNTX has also been shown to act directly on the muscle by producing contractures in the mouse hemidiaphragm in the presence of tubocurarine. Furthermore, blockade of the SNTX-induced contractures by dantrolene sodium, which causes blockade prior to activation of the contractile proteins in the muscle, indicates SNTX does not act directly on the contractile muscle proteins [113]. This is further evidence that *Synanceia* venom is myotoxic and likely to induce the permanent damage observed by electron microscopy at the neuromuscular apparatus in skeletal muscle [113,114].

Recent research on crude fresh and lyophilized venom from *S. verrucosa* investigated the activity of the venom on mimotopes of nicotinic acetylcholine receptors (nAChRs) from four different taxa [47]. The fresh venom was able to bind to all mimotopes, whereas the lyophilized venom was significantly reduced in activity [47]. This binding action indicates that *S. verrucosa* venom might act on postsynaptic nAChRs, agreeing with previous research on chick biventer cervicis nerve preparations using *S. horrida* venom [48]. Interestingly, this difference between the activities of lyophilized and fresh venoms could be due to the size of the toxins responsible for the nAChR binding, likely being labile and large [47].

Further testing on mammalian L-type Ca_V_1.2 channel mimotopes of domains I–IV (DI–DIV) using biolayer interferometry (BLI) showed that both lyophilized and fresh venoms bound only to DIV, indicating that the toxins responsible for this particular activity might be small peptides or an amine-type molecule, which are more stable than proteinaceous or enzymatic molecules [47]. These results also support previous research that suggests the activation of Ca^2+^ channels by *Synanceia* venom [62,65]. Unfortunately, it was not possible to verify by BLI if the Ca^2+^ channel activity of the venom acts as an antagonist or agonist [47], and so, there is still much to be studied.

In addition, SNTX promotes the secretion of catecholamine from neuroendocrine cells through a Ca^2+^-dependent exocytosis of large dense-core vesicles in the presence of extracellular Ca^2+^ in bovine chromaffin cells [115]. This soluble *N*-ethylmaleimide-sensitive fusion protein attachment protein receptor (SNARE)-dependent exocytosis was shown to be independent of Ca^2+^ influx through voltage-activated Ca^2+^ channels, as blockade of the L-, N- and P/Q-type channels resulted in a minimal change in catecholamine secretion [115]. Intracellular fluorescence experiments also showed that SNTX-induced catecholamine secretion required sufficient internal stores of Ca^2+^, as depletion of intracellular Ca^2+^ with caffeine, thapsigargin or ryanodine resulted in a reduced SNTX-induced catecholamine response [115].

Many of the effects of *Synanceia* venom have a time- and dose-dependent relationship. *S. horrida* venom, for example, causes permanent depolarization of muscle fibers at high concentrations, whereas at low concentrations it causes massive release and depletion of neurotransmitters in frog nerve–muscle preparations [114]. Additionally, high concentrations of venom cause both muscle and nerve damage, with nerve terminal swelling and no synaptic vesicles evident. These effects, combined with the observation of a total absence of miniature endplate potentials following prolonged periods of high-frequency potentials, indicate *Synanceia* venom also blocks the recycling of synaptic vesicles [114]. When comparing *Synanceia* venom to other animal toxins with similar neuromuscular blocking activity via the release and depletion of neurotransmitters from the nerve terminal, such as α-latrotoxin from the spider genus *Latrodectus* [116], *Synanceia* venom is differentiated by also causing muscle depolarization and microscopic muscle damage. An exception to this is the pardaxins, toxins found in the dorsal and anal fin gland secretions of the Pacific sole (*Pardachirus pavoninus*) and Red Sea Moses sole (*P. marmoratus*). *Synanceia* venom mimics the action of the pardaxins, acting as a presynaptic neurotoxin at low concentrations and severely damaging skeletal muscle fibers at high concentrations [117,118]. Interestingly, although both *Synanceia* venom and the pardaxins lyse dog erythrocytes, only the pardaxins lyse human erythrocytes [114,119].

Stonefish venom-elicited release of neurotransmitters has been reported not to involve voltage-gated sodium channels (Na_V_) [114]. This was determined from experiments where sodium channel currents blocked by tetrodotoxin (TTX) did not affect venom-elicited neurotransmitter release. It is, however, possible that neurotransmitter release may involve the TTX-resistant Na_V_1.8 [120] or Na_V_1.9 [121] sodium channel subtypes, both discovered subsequent to the 1993 study of Kreger et al. Activation of these channel subtypes is associated with pain [122] and is consistent with the symptoms of stonefish envenomation.

## 9. Conclusions and Future Directions

Stings caused by venomous fish species occur worldwide. While the causative agent of many of these stings is considered to be “stonefish”, it should be noted that the identity of the offending animal cannot be confirmed in most cases. As a result, reports of stonefish stings should be viewed with caution when the animal has not been captured. Notably, individuals belonging to the genus *Synanceia* are widely distributed, and while stonefish cause serious envenomations worldwide, the extent of involvement of other venomous fish species in serious envenomations around the globe is not fully understood.

Studies investigating the pathophysiological mechanisms and effects of *Synanceia* venom at the cellular, tissue and organ levels have significantly advanced since the 1990s. Of note, all published work on *Synanceia* venom has been performed only on two species thus far: *S. horrida* and *S. verrucosa*. Characterizing the venom of additional species for future research is likely to increase understanding of the pathophysiological mechanisms and effects of stonefish venom.

Of note, while the cytolytic activity of stonefish venom appears to be the result of pore-forming action on cell membranes, the precise mode of action of the venom and its individual components still remains largely unknown. It has become clearer that the biological symptoms and pathologies observed in sting victims cannot solely be attributed to the presence of a single venom protein, such as SNTX or VTX, as initially thought. Instead, these effects are likely a result of the combined activity of the many enzymes and proteins that have now been discovered in *Synanceia* venom, many of which appear to be novel. Indeed, only one of these enzymes, hyaluronidase, has been characterized on a molecular level, and yet its role in the envenomation effects remains poorly understood. Although the rich bioprospecting potential of *Synanceia* venoms has yet to be realized, the results of recent transcriptomic- and proteomic-based approaches to venom characterization highlight the need for more research into these medically important fish species. The study of *Synanceia* venom variability, composition and immunological cross-reactivity may also have a direct impact on human health by contributing to the development of improved antivenoms, thereby reducing the burden of venomous fish species on human health worldwide.

## Figures and Tables

**Table 1:** In vivo and in vitro cross-reactivity studies between venomous fish species and *Synanceia* antivenom.

Fish Species	Neutralizing Action	Reference
*Synanceia verrucosa*	Lethal and hemolytic	[35]
*Inimicus japonicus*	Lethal and hemolytic	[35]
*Pterois lunulata*	Lethal and hemolytic	[35]
*Pterois antennata*	Lethal and hemolytic	[35]
*Dendrochirus zebra*	Lethal and hemolytic	[35]
*Pterois volitans*	Lethal, hemolytic and pharmacological	[35,42]
*Gymnapistes marmoratus*	Pharmacological	[42]
*Scorpaena plumieri*	Inflammatory and cardiovascular	[43]
*Notesthes robusta*	No effect	[44]

**Table 2:** Toxins found in stonefish venom with their corresponding molecular weights (MW).

*Synanceia* Species	Toxins	MW (kDa)	Subunit MW (kDa)	Reference
***S. horrida***	SNTX	148	α-subunit—79	[57]
			β-subunit—79	
	Cytolysin	158	-	[58]
	SFHYA1	62	-	[59]
	Peroxiredoxin-6	24	-	[60]
***S. verrucosa***	VTX	322	2x α-subunit—83	[49]
			2x β-subunit—78	
	NeoVTX	166	α-subunit—79	[61]
			β-subunit—79	
	Cardioleputin	46	-	[62]
	Con A-I-PS-I	42.1	-	[63]
		100		
		110		
	45 kDa lectin	45	-	[63]
	Hyaluronidase	59	-	[64]

**Table 3:** LD_50_ values in mice from two different species of *Synanceia* venoms.

*Synanceia* Species	Toxic Component	Route	LD_50_ (µg/kg)	Reference
***S. horrida***	Crude venom	IV	0.4–0.6	[67]
	Reconstituted venom	IV	220	[68]
			300–666	[45,56,69]
		SC	2666–4000	[45]
		IC	266	[45]
		IP	1333–2000	[45,58]
	Fraction 1	IV	35	[56]
	SNTX	IV	17	[56]
***S. verrucosa***	Crude venom	IV	360	[70]
			180	[35]
			125 (estimated)	[49]
	Crude venom	IM	107	[71]
			38	[72]
	NeoVTX	IV	47	[70]

IC–intracerebral; IM—intramuscular; IP—intraperitoneal; IV—intravenous; SC—subcutaneous.

## Appendix A

**Table A1:** List of case reports where stonefish from the genus *Synanceia* have been reported as the culprit for human envenomations. As identification of the animal is difficult, caution should be used when stating the causative agent of hospitalizations, as envenomation may have been caused by another venomous fish found in the region.

Location	Number of Cases	Year	Reference
**Australia**			
Cairns	1		[123]
Cooktown	1		[123]
Western Australia	1		[124]
Tropical northern Australia	1		[125]
Cook Islands	1		[31]
**Egypt**			
Taba	1		[126]
**French Polynesia**			
Bora Bora	1	2018	[27]
Tubuai Island	1	2014	[27]
Guam	2		[99,127]
**Indonesia**			
Lembeh, North Sulawesi	1		[128]
Indo-Pacific region	1		[32]
**Japan**			
Okinawa	15	2013–2017	[6]
**Kingdom of Bahrain**	1		[30]
**Malaysia**			
Kota Kinabalu, Sabah	1		[129]
**Mozambique**			
Pinda	1	1956	[34]
New Caledonia	1	2008	[27]
**Papua New Guinea**			
Trobriand Islands	12		[130]
**People’s Republic of China**			
Hong Kong	1	2002	[131]
Hong Kong	1	2003	[131]
Hong Kong	7	2005–2008	[5]
Not clearly stated—possibly Hong Kong	1	2008	[28]
Hong Kong	32	2008–2018	[4]
Not clearly stated	1		[26]
**Seychelles**			
Pont Larue, Mahe	1	1956	[34]
**Singapore**			
Pulau Bukom	81	4 years	[25]
Location not stated	1	2001–2003	[11]
Location not stated	7	1.25 years	[11]
Location not stated	30	2004–2006	[9]

**Table A2:** Geographical locations of *Synanceia* specimens throughout the globe. Coordinates are shown as they were presented in the literature.

Location per Continent	Number of Specimens	Coordinates	Reference
*S. verrucosa*			
**AFRICA**			
Kenya–Andromache Reef, S of Port Kilindini of Mombasa Harbor	1	4°05′ 05″ S, 39°40′ 39″ E	[2]
Mauritius—location not stated	1		[2]
Mozambique—Pinda Peninsula	1	14°14°12″ S	[132]
Seychelles—location not stated	2		[2]
**ASIA**			
Ceylon—Trincomalee, inside base of Royal Navy of Ceylon	3		[2]
Cyprus—Kumyali	1		[133]
Japan—Okinawa	1		[2]
**Mediterranean Sea**			
Gaza City, State of Palestine	1	31°31′ 3.32″ N, 34°25′ 18.66″ E	[134]
Israel—Palmakhim	1	31°56.36′ N, 34°42.16′ E	[135]
Lebanon—Tyr	1	33.290657° N, 35.184459° E	[136]
Syria—Lattakia city	1	35°31.5′ 5.97″ N, 35°42′ 48.57″ E	[137]
Turkey—Yumurtalik, Iskenderum Bay	1		[138]
**Phillipines**			
Dumaguette	1		[2]
Sitankai, Sulu Province	2		[2]
Location not stated	1		[2]
**Red Sea**			
Location not stated	1		[2]
Thailand—Ko Tao, Sairee Beach, Gulf of Thailand	1		[139]
**Western Indian Ocean**			
Southern Andaman Islands, S of Corbyn’s Cove, Port Blair	1		[2]
**OCEANIA**			
**Australia**			
Capricorn Islands, One Tree Island, W side, QLD	1		[2]
Exmouth, WA	1	−21.958648, 114.141082	[140]
Fairfax Island, QLD	1		[2]
Gulf of Carpentaria, QLD	1	15°1′ 30″ S, 138°41′ 30″ E	[141]
Heron Island, QLD	1		[13]
Lancelin, WA	1	31°0′ S, 115°19′ E	[142]
Magnetic Island, Townsville, QLD	1	19°08′ 00″ S, 146°50’00ʺ E	[143]
Mermaid Reef, Rowley Shoals, WA	1	−17.083, 119.583	[144]
Shark Bay, New Beach, 45 km S of Carnarvon	1	25°20′ S, 113°56′ E	[145]
Tallow Beach, 300 m S of the southern boundary of Arakwal NP, NSW	1	28°39′ 54″ S, 153°37’ 32″ E	[146]
**Melanesia**			
*Solomon Islands*			
Bougainville, E side of Puk Puk Island, outside Poison Lagoon	1		[2]
Sikaiana Island	1		[2]
**Micronesia**			
*Caroline Islands*			
Yap Island, inlet E side of Yap Island	1	9°29′ 48″ N, 138°26′ 57″ E	[2]
Location not stated	4		[2]
*Mariana Islands*			
Guam, Hagatna	1		[2]
Guam, N of Tringhera Beach in Hagatna Bay	3	13°28′ 53″ N, 144°45′ 45″ E	[2]
Guam, SW of Agat village, N side of Bangi Point	1	13°22′ 36″ N, 144°38′ 53″ E	[2]
Location not stated	3		[2]
*Palau Islands*			
Angaur Island, in Garangaoi Cove, S of Cape Nagaramudel	1	6°53′ 50″ N, 137°7′ 49″ E	[2]
Auluptagel Island, Crocodile Cove	1	7°17′ N, 137°29′ E	[2]
Ngadarak Reef SW of Auluptagel Island	2	7°17′ 48″ N, 134°28′ 37″ E	[2]
Location not stated	5		[2]
New Caledonia—Noumea	1		[2]
New Guinea—location not stated	1		[2]
**Polynesia**			
*Society Islands*			
Moorea, Faatoai village at Papetoai Bay	1		[2]
**Tahiti**	1		[2]
Location not stated	4		[2]
*Fiji*			
Location not stated	1		[2]
*Samoa*			
Pago Pago	2		[2]
Location not stated	1		[2]
Location not stated	4		[2]
*Tonga Islands*			
Location not stated	1		[2]
*Tuamotu Islands*			
Location not stated	3		[2]
***S. horrida***			
**ASIA**			
Batavia (Jakarta)	1		[2]
Malasia—location not stated			[147]
**Phillipines**			
Atimonan, Tayabas	1		[2]
Calaogao, Cauayan, Negros Island	1	10°N, 122°30′ E	[2]
Manila Bay	1		[2]
Ragay Gulf, Luzon	1		[2]
Stankai, Sulu Island	1		[2]
Location not stated	2		[2]
**Singapore**			
Pacific Expedition—location not stated	5		[2]
Punggol—location not stated	1		[25]
Singapore Market	1		[2]
Location not stated	1		[2]
**Thailand**			
Patong Bay, Patong Phuket	2		[2]
Rayong Province, SE of Ban Phe Fisheries Station	1	12°35′ 40″ N, 101°25′43″ E	[2]
Location not stated	1		[2]
**OCEANIA**			
New Guinea—Waigeo Island	1		[2]
**Australia**			
Between Moreton Bay and Cairns, Queensland, Australia	52		[13]
Britomart Reef, QLD	1	18°10′ S, 146°43′ E	[148]
Coffs Harbor, NSW	1	30°15′ S, 153°8′ E	[149]
East coast of Northern Queensland	25-30		[150]
Melville Bay and Cape Arnhem area, NT	1	12°15′ S, 136°43′ E	[151]
Moreton Bay			[13]
N side of Main Wharf, Broome, WA	1	−17.967, 122.233	[152]
Northern Territory, Groote Eylandt	5		[2]
Port Darwin, NT	1	12°27′ S, 130°48′ E	[153]
Port Hedland, WA	1	20°18′ S, 118°35′ E	[154]
Princess Charlotte Bay, QLD	1	−14.333333, 144.116667	[155]
Shark Bay, blow holes, N of Carnarvon	1	−24.483333, 113.416667	[156]
Sweers Island, Gulf of Carpentaria, QLD	1	17°6′ S, 139°37′ E	[157]
Tryon Island, Capricorn Group, QLD	1	23°15′ S, 151°47′ E	[158]
***S. nana***			
**ASIA**			
Pakistan—Karachi Fish Harbor	1		[159]
**Red Sea**			
Gulf of Suez—Et-Tur, Sinai Peninsula	1		[2]
Gulf of Suez—off Port Safaga	1	27°16′ 15″ N, 33°47′ 30″ E	[2]
Israel—Gulf of Aqaba, between Marset Mahash el Ala and Marset Abu Samra	1		[2]
Israel—NW coast of the Gulf of Aqaba, bay at Al Himeira	5		[2]
Saudi Arabia—Persian Gulf, Tarut Bay, Near Ras Tanura spit	1		[2]
***S. alula***			
**OCEANIA**			
Nicobar Islands—Nancowry Island	2	8°N, 93°40′ E	[2]
Solomon Islands—New Georgia, Munda Lagoon	1		[2]
Solomon Islands—New Georgia, Munda Pier	2		[2]
***S. platyrhyncha***			
**ASIA**			
Ambon Island	1		[2]
